# In the national epidemiological bulletins – a selection from current issues

**DOI:** 10.2807/1560-7917.ES.2016.21.44.30387

**Published:** 2016-11-03

**Authors:** Eurosurveillance editorial team

**Affiliations:** 1 European Centre for Disease Prevention and Control (ECDC), Stockholm, Sweden

**Keywords:** public health

**Respiratory diseases**
Legionella pneumonia 2015
EPI-NEWS 42/43, 2016
26 October 2016, Denmark

http://www.ssi.dk/English/News/EPI-NEWS/2016/No%2043%20-%202016.aspx

Enhanced Surveillance of Mycobacterial Infections (ESMI) in Scotland: 2016 tuberculosis annual report for Scotland
HPS Weekly Report, 2016;50(43)
25 October 2016, Scotland

http://www.hps.scot.nhs.uk/documents/ewr/pdf2016/1643.pdf

*Mycoplasma pneumoniae* epidemic
EPI-NEWS 41, 2016
12 October 2016, Denmark

http://www.ssi.dk/English/News/EPI-NEWS/2016/No%2041%20-%202016.aspx

Middle East respiratory coronavirus (MERS-CoV). Monitoring of suspected cases in France, October 2012– December 2015
Bulletin épidémiologique hebdomadaire, 32–33
11 October 2016, France

http://invs.santepubliquefrance.fr/beh/2016/32-33/pdf/2016_32-33.pdf

Influenza activity in mainland France, season 2015–2016
Bulletin épidémiologique hebdomadaire, 32–33
11 October 2016, France
http://invs.santepubliquefrance.fr/beh/2016/32-33/pdf/2016_32-33.pdf

**Food- and waterborne diseases**
VTEC in Scotland 2015: enhanced surveillance, reference laboratory and clinical reporting data
HPS Weekly Report, 2016;50(42)
18 October 2016, Scotland
http://www.hps.scot.nhs.uk/documents/ewr/pdf2016/1642.pdf
Salmonella trends in humans, farm animals and food
Infectieziekten Bulletin 2016; 27(8)
October 2016, the Netherlands
http://www.rivm.nl/Documenten_en_publicaties/Algemeen_Actueel/Uitgaven/Infectieziekten_Bulletin/Jaargang_27_2016/Oktober_2016/Inhoud_oktober_2016/Trends_in_Salmonella_bij_de_mens_landbouwhuisdieren_en_in_voedsel
Trichinellosis outbreak after eating wild boar meat in Limburg and Antwerp restaurants in December 2014
Vlaams Infectieziektebulletin; 3/2016
October 2016, Belgium
https://www.zorg-en-gezondheid.be/sites/default/files/atoms/files/2016-3-Trichinellose-outbreak-%20A.forier.pdf

**Vaccine-preventable diseases**
Invasive meningococcal disease in Germany 2012 – 2015
Epidemiologisches Bulletin 43, 2016
31 October 2016
https://www.rki.de/DE/Content/Infekt/EpidBull/Archiv/2016/Ausgaben/43_16.pdf?__blob=publicationFile
The epidemiology of human papilloma virus in women aged 20–65 years living in remote villages in French Guiana: Adapting interventions to the territory
Bulletin épidémiologique hebdomadaire, 34
18 October 2016, France
http://invs.santepubliquefrance.fr/beh/2016/34/pdf/2016_34.pdf
Cases of pertussis on the rise this autumn
Folkhälsomyndigheten website
12 October 2016, Sweden
https://www.folkhalsomyndigheten.se/nyheter-och-press/nyhetsarkiv/2016/oktober/fler-fall-av-kikhosta-i-host/
Rubella screening of pregnant women by midwives
Infectieziekten Bulletin 2016; 27(8)
October 2016, the Netherlands
http://www.rivm.nl/Documenten_en_publicaties/Algemeen_Actueel/Uitgaven/Infectieziekten_Bulletin/Jaargang_27_2016/Oktober_2016/Inhoud_oktober_2016/Rubellascreening_bij_zwangere_vrouwen_door_verloskundigen

**Sexually transmitted diseases**
HIV infection and AIDS: Quarterly report to 30 June 2016
HPS Weekly Report, 2016;50(41)
11 October 2016, Scotland
http://www.hps.scot.nhs.uk/documents/ewr/pdf2016/1641.pdf

**Zoonoses and vector-borne diseases**
Zika virus infection in a traveller returning from Vietnam
Epidemiologisches Bulletin 42, 2016
24 October 2016
https://www.rki.de/DE/Content/Infekt/EpidBull/Archiv/2016/Ausgaben/42_16.pdf?__blob=publicationFile
Initial Danish experiences with Zika virus Diagnostics
EPI-NEWS 41, 2016
12 October 2016, Denmark
http://www.ssi.dk/English/News/EPI-NEWS/2016/No%2041%20-%202016.aspx
Chikungunya and dengue surveillance in mainland France, 2015
Bulletin épidémiologique hebdomadaire, 32–33
11 October 2016, France
http://invs.santepubliquefrance.fr/beh/2016/32-33/pdf/2016_32-33.pdf
Rabies in Belgium, a potential danger? One case
Vlaams Infectieziektebulletin; 3/2016
October 2016, Belgium
https://www.zorg-en-gezondheid.be/sites/default/files/atoms/files/2016-3-Rabies-B-casus-F.Hoefkens.pdf

**Other**
No urban myth: reception of monkeys with herpes B virus infection
Infectieziekten Bulletin 2016; 27(8)
October 2016, the Netherlands
http://www.rivm.nl/Documenten_en_publicaties/Algemeen_Actueel/Uitgaven/Infectieziekten_Bulletin/Jaargang_27_2016/Oktober_2016/Inhoud_oktober_2016/Geen_broodjeaapverhaal_opvang_van_apen_met_herpes_B_virusinfectie

